# MiR‐18a‐5p Attenuates Oxidative Stress and Inhibits Lipid Accumulation in Alcoholic Fatty Liver by Activating the CYP1A1‐PPAR Axis

**DOI:** 10.1002/iid3.70350

**Published:** 2026-02-05

**Authors:** XueMei Zhang, Wei Li, Ubaid Ullah, Ning Li, XinYu Geng, JiHan Qi, HongLiang Chen, Xu Zhang, Ying Hu, Lingling Yang, ShiZhu Jin

**Affiliations:** ^1^ Department of Gastroenterology and Hepatology The Second Affiliated Hospital of Harbin Medical University Harbin China; ^2^ Department of Gastroenterology The First Affiliated Hospital of Jiamusi University Jiamusi China

**Keywords:** alcoholic fatty liver disease, CYP1A1, lipid metabolism, miR‐18a‐5p, Oxidative stress, PPAR

## Abstract

**Background:**

Lipid deposition in alcoholic fatty liver disease (AFL) represents an early stage in alcoholic liver disease progression and may contribute to carcinogenesis. MicroRNAs (miRNAs) play critical regulatory roles in liver biological processes.

**Methods:**

In this study, we explored the miR‐18a‐5p/CYP1A1/PPAR axis in AFL using bioinformatics approaches. An AFL rat model was created, and second‐generation sequencing identified differentially expressed mRNA in rat liver tissues. Core genes were identified through Gene Ontology, Kyoto Encyclopedia of Genes and Genomes pathway, and Gene Expression Omnibus database analyses. These genes were validated by qPCR in liver tissues and via in vitro experiments using L02 cells. The upstream miRNA identified in AFL was further verified in L02 cells using luciferase reporter assays.

**Results:**

Differential gene analysis revealed CYP1A1 and the PPAR pathway. In alcohol‐induced L02 cells, increased CYP1A1 expression promoted oxidative stress and altered lipid metabolism via the PPAR pathway. MiR‐18a‐5p was identified as an upstream regulator that targets CYP1A1 to ameliorate alcohol‐induced oxidative stress. Inhibition of CYP1A1 by miR‐18a‐5p improved the expression of PPARγ‐related genes and decreased PPARα‐related gene expression, thereby reducing lipid deposition.

**Conclusion:**

The miR‐18a‐5p/CYP1A1/PPAR axis is a novel pathway that could be targeted for AFL treatment.

## 1 Introduction

The liver, as the primary organ responsible for alcohol metabolism [1], is highly susceptible to damage from chronic excessive alcohol consumption, which can lead to alcoholic liver disease (ALD). ALD progresses through distinct stages, including alcoholic fatty liver (AFL), steatohepatitis, hepatitis, liver fibrosis, cirrhosis, and potentially liver cancer [2, 3, 4]. With approximately 75 million people globally suffering from alcohol dependence [5], and persistent alcohol exposure activates hepatic immune cells (e.g., Kupffer cells), triggering pro‐inflammatory cytokine (tumor necrosis factor‐α [TNF‐α], interleukin‐6 [IL‐6]) release, which exacerbates hepatocellular damage and disease progression to cirrhosis or cancer [6, 7], early‐stage ALD such as AFL remains poorly characterized due to the lack of specific biomarkers, often leading to underdiagnosis and underscoring the urgent need to unravel its underlying mechanisms. Although the advanced stages of ALD have been extensively studied, AFL, as an early manifestation of ALD, still lacks effective diagnostic biomarkers, and its pathogenesis has not yet been fully elucidated. In particular, there is a lack of in‐depth investigation into lipid metabolism and oxidative stress, especially with respect to miRNA‐mediated molecular networks.

MicroRNAs (miRNAs) emerged as key modulators of liver metabolism and immune responses. They are key regulators of mRNA stability and protein expression, and their dysregulation has been extensively linked to the pathogenesis of various liver diseases, including ALD [8, 9, 10, 11]. Among them, miR‐18a‐5p, as a miRNA with potential regulatory functions, may play an important role in the pathogenesis of AFL. Among the molecular pathways involved in hepatic lipid metabolism and oxidative stress‐hallmark features of AFL‐the cytochrome P450 1A1 (CYP1A1) and peroxisome proliferator‐activated receptor (PPAR) axis has garnered increasing attention. Recent studies have highlighted CYP1A1's role in mediating alcohol‐induced oxidative stress [12, 13], while PPARs are critical regulators of lipid homeostasis, with their dysregulation closely associated with hepatic steatosis in both alcoholic and nonalcoholic contexts [14, 15, 16]. However, the interplay between CYP1A1 and PPARs in AFL, particularly how miR‐18a‐5p functions within this pathway, remains unclear and requires further investigation. Clarifying this will be of great significance for filling this knowledge gap and elucidating the early molecular mechanisms of AFL.

Against this backdrop, the present study hypothesizes that the miR‐18a‐5p/CYP1A1/PPAR axis plays a pivotal role in AFL pathogenesis by regulating oxidative stress and lipid deposition. Specifically, we aim to: identify differentially expressed genes and miRNAs associated with AFL using bioinformatics and experimental models; validate the regulatory relationship between miR‐18a‐5p, CYP1A1, and PPARs; and clarify the mechanistic role of this axis in AFL development. This work fills a critical gap by linking miRNA‐mediated regulation of CYP1A1 to PPAR‐dependent metabolic pathways, thereby identifying a novel therapeutic target for AFL.

## 2 Results

### 2.1 Lipid Deposition and RNA‐Seq Analysis in AFL Rat Liver

**Figure 1:**
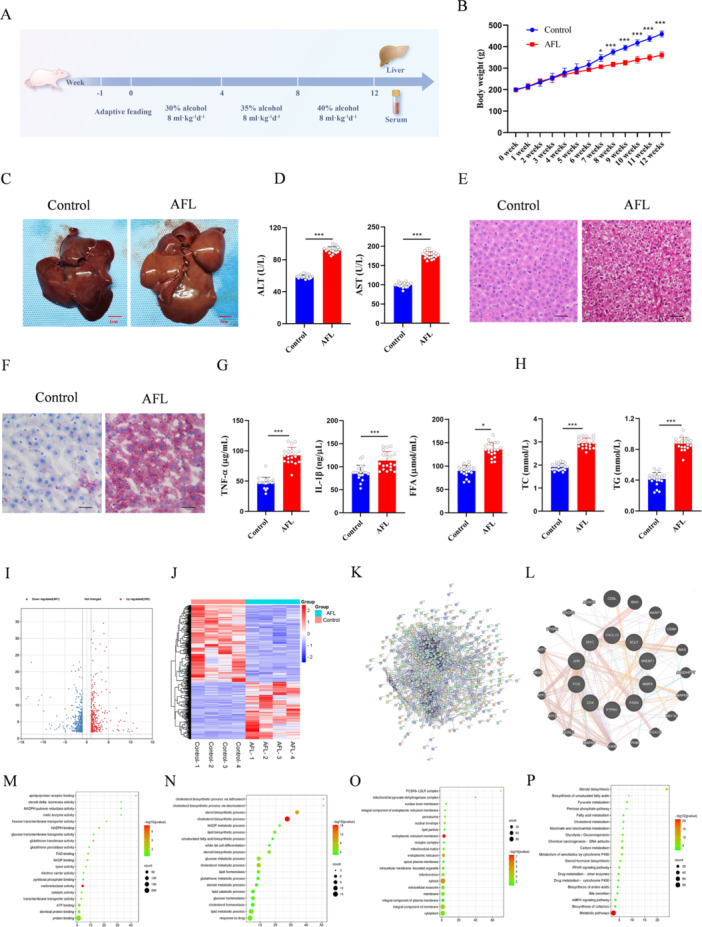
Establishment of the AFL animal model and RNA‐seq analysis. (A) Schematic representation of the process of establishing the AFL rat model and serum analysis. The control group (*n* = 10) and the alcohol‐fed group (*n* = 10). (B) Growth curves of body weight in rats from the NC (normal control) and AFL groups. (C) Macroscopic specimens of rat livers from the NC and AFL groups. (D) ELISA analysis of serum ALT and AST levels in rats from the NC and AFL groups. (E, F) H&E and Oil Red O staining of rat liver tissues, magnification 200×; scale bar, 100 µm. (G) ELISA analysis of serum TNF‐α and IL‐1β levels in rats from the NC and AFL groups. (H) Measurement of serum FFA, TC, and TG levels in rats from the NC and AFL groups. (I) Volcano plot of differentially expressed genes (|LogFC | ≥ 1 *p* < 0.05). (J) Heat map of differentially expressed genes. (K) Protein‐protein interaction analysis of differentially expressed genes. (L) Core protein sub‐network constructed using the GeneMANIA database. (M–P) GO enrichment analysis of differentially expressed genes and KEGG enrichment analysis. *n* = 6, *p < 0.05, ***p < 0.001.

An AFL rat model was developed using a gradient alcohol gavage method (Figure 1A). Compared to controls, AFL rats showed reduced weight gain (Figure 1B), liver enlargement (Figure 1C), increased serum ALT and AST levels (Figure 1D), liver tissue vacuolar changes (Figure 1E,F), and elevated serum TNF‐α, IL‐1β, FFA, TC, and TG levels (Figure 1G,H). Transcriptome sequencing revealed 839 differentially expressed genes (Figure 1I–L), with enrichment in lipoprotein binding, oxidoreductase activity, and metabolic processes. Key pathways included cytochrome P450 metabolism, ketone body metabolism, fatty acid metabolism, cholesterol metabolism, and the PPAR signaling pathway (Figure 1M–P).

### 2.2 Alcohol‐Induced Expression of CYP1A1 and Oxidative Stress

**Figure 2:**
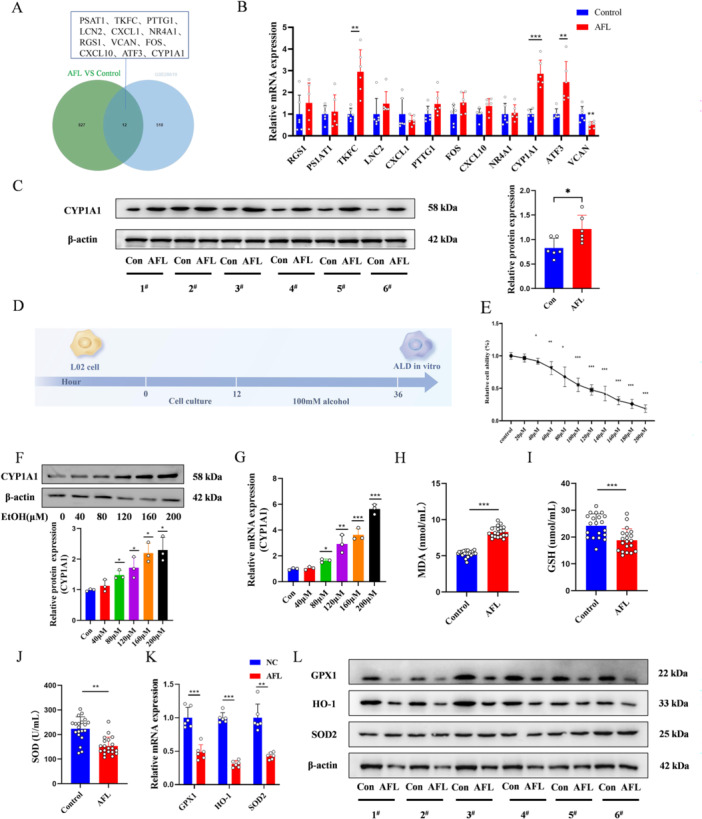
Alcohol‐Induced Increase in CYP1A1 Expression in the Liver. (A) Venn diagram showing the common differentially expressed genes between RNA‐Seq and GSE28619. (B) qPCR validation of 12 differentially expressed genes. (C) Western blot analysis of CYP1A1 protein levels in liver tissues of control and AFL groups. (D) CCK‐8 assay to assess the impact of different alcohol concentrations on L02 cell viability. (E) Schematic representation of the establishment of the cell AFL model. (F) Western blot analysis of changes in CYP1A1 protein levels with different alcohol concentrations. (G) qPCR analysis of changes in CYP1A1 mRNA with different alcohol concentrations. (H–J) Kit assay to measure MDA, GSH, and SOD levels in serum of AFL rat models. (K) qPCR analysis of oxidative stress‐related genes in rat liver tissues. (L) Western blot analysis of oxidative stress‐related proteins in rat liver tissues. *n* = 3, *p < 0.05, **p < 0.01, ***p < 0.001.

Gene expression profiles from the GEO database for alcoholic hepatitis and normal liver tissues identified 12 significantly altered genes (Figure 2A), CYP1A1 is one of the genes with upregulated expression (Figure 2B,C). CCK‐8 assay demonstrated a dose‐dependent decrease in cell viability with increasing alcohol concentration. The IC50 value for L02 cells exposed to alcohol was 104.23 ± 3.77 μM, 100 μM alcohol was selected to simulate the AFL model in vitro (Figure 2D,E). Western blot and qPCR validation showed upregulation of CYP1A1 in AFL rat livers and L02 cells treated with alcohol, indicating alcohol‐induced CYP1A1 expression (Figure 2F,G). Increased MDA levels and decreased GSH and SOD levels in AFL rats confirmed oxidative stress induction (Figure 2H–J). GPX1, HO‐1, and SOD2 significantly decreased after alcohol treatment (Figure 2K,L).

### 2.3 CYP1A1 Regulates Lipid Deposition and Oxidative Stress via the PPAR Pathway

**Figure 3:**
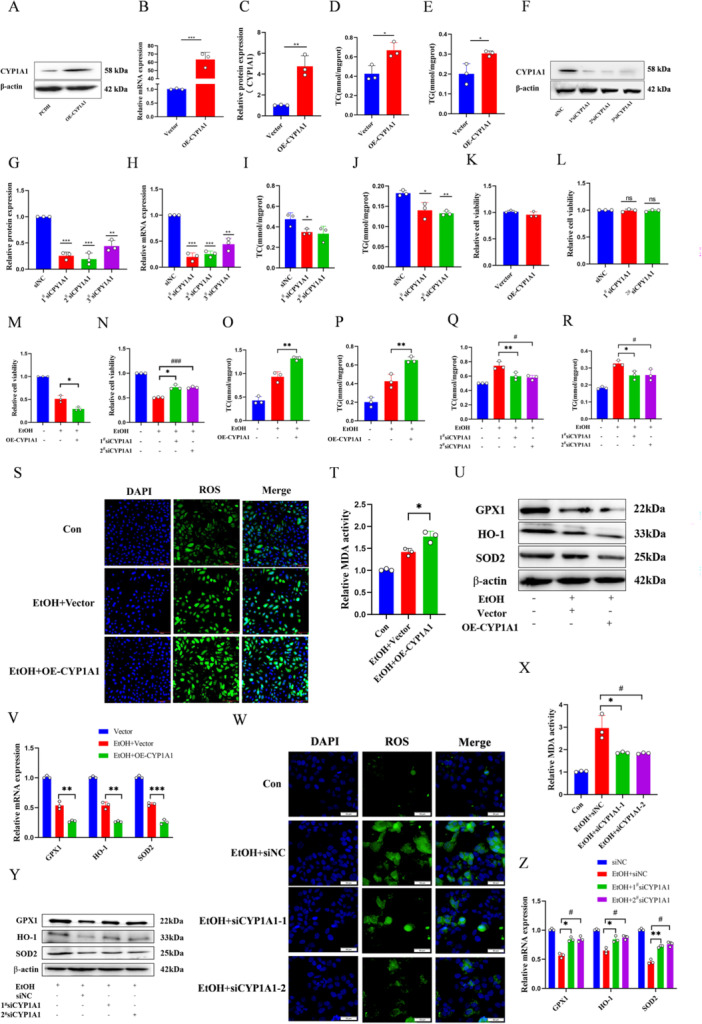
CYP1A1 aggravates alcohol‐induced oxidative stress and lipid deposition in L02 cells. (A) Western blot analysis of CYP1A1 protein expression in L02 cells. (B, C) Impact of CYP1A1 overexpression on TC and TG levels in L02 cells. (D) qPCR analysis of CYP1A1 mRNA expression in L02 cells. (E) Analysis of CYP1A1 protein grayscale after overexpression. (F) Western blot analysis of siRNA effects on CYP1A1 protein expression. (G, H) Impact of CYP1A1 knockdown on TC and TG levels in L02 cells. (I) qPCR analysis of siRNA effects on CYP1A1 mRNA expression in L02 cells. (J) Analysis of CYP1A1 protein grayscale after knockdown. (K, L) CCK‐8 assay to assess the impact of CYP1A1 overexpression and interference on cell viability. (M, N) CCK‐8 assay to assess the impact of CYP1A1 overexpression and interference on alcohol‐induced L02 cell viability. (O–R) Impact of CYP1A1 overexpression or interference on alcohol‐induced TC and TG levels in L02 cells. (S) DCFH‐DA probe detection of ROS levels in alcohol‐induced L02 cells after CYP1A1 overexpression (scale bar = 100 μm). (T) Impact of CYP1A1 overexpression on MDA levels in alcohol‐induced L02 cells. (U) Western blot analysis of the effects of CYP1A1 overexpression on oxidative stress‐related protein levels in alcohol‐induced L02 cells. (V) qPCR analysis of the effects of CYP1A1 overexpression on oxidative stress‐related mRNA levels in alcohol‐induced L02 cells. (W) DCFH‐DA probe detection of ROS levels in alcohol‐induced L02 cells after CYP1A1 knockdown (scale bar = 50 μm). (X) Impact of CYP1A1 knockdown on MDA levels in alcohol‐induced L02 cells. (Y) Western blot analysis of the effects of CYP1A1 knockdown on oxidative stress‐related mRNA and protein levels in alcohol‐induced L02 cells. (Z) qPCR analysis of the effects of CYP1A1 interference on oxidative stress‐related mRNA levels in alcohol‐induced L02 cells. *n* = 3, *p < 0.05, **p < 0.01, ***p < 0.001, ^#^ *p* < 0.05, ^###^ *p* < 0.001.

CYP1A1 overexpression in L02 cells increased TC and TG levels and promoted oxidative stress under alcohol stimulation (Figure 3A–E), but CYP1A1 knockdown had the opposite effects (Figure 3F–J). While CYP1A1 had no effect on L02 cell viability under normal culture conditions (Figure 3K,L), its overexpression promoted the inhibitory effect of alcohol on cell viability under alcohol stimulation, partially relieved by knocking down CYP1A1 (Figure 3M–O). Furthermore, CYP1A1 overexpression facilitated the alcohol‐induced increase in TG and TC levels, while knocking down CYP1A1 inhibited these indicators (Figure 3P–R). Overexpression of CYP1A1 increased alcohol‐induced reactive oxygen species (ROS) and MDA levels (Figure 3S,T), Consistent with this conclusion, CYP1A1 overexpression further enhanced the inhibitory effect of alcohol on the expression of oxidative stress‐related molecules GPX1, HO‐1, and SOD2 (Figure 3U,V). Knocking down CYP1A1 suppressed ROS and MDA levels (Figure 3W,X), alleviating the inhibitory effect of alcohol on GPX1, HO‐1, and SOD2 expression (Figure 3Y,Z). In conclusion, CYP1A1 overexpression increased TC, TG, and oxidative stress in L02 cell under alcohol stimulation, inhibits cell viability, and exacerbates the elevation of ROS and MDA levels. Conversely, knocking down CYP1A1 alleviates these adverse effects.

**Figure 4:**
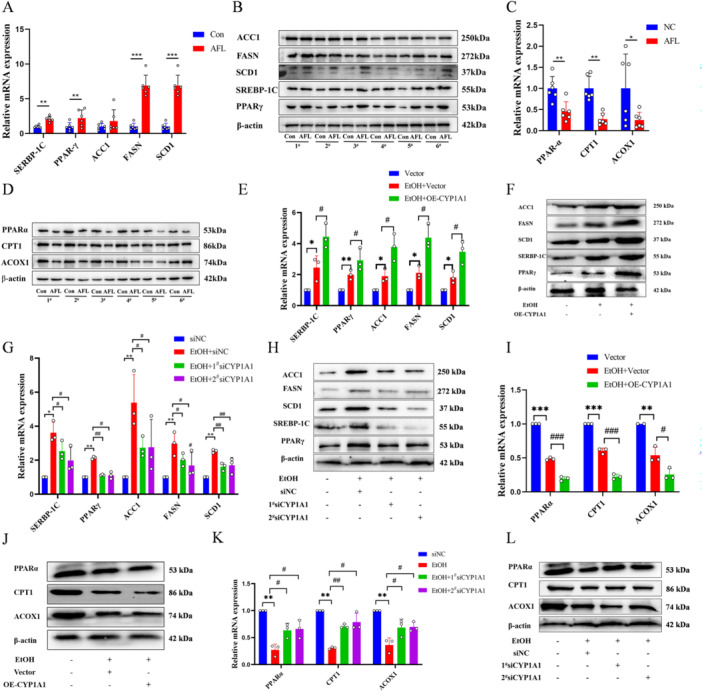
Impact of CYP1A1 on alcohol‐induced expression of PPAR‐related genes in L02 cells. (A–D) qPCR and Western blot analysis of PPARγ‐related (A‐B) and PPARα‐related (C, D) gene mRNA and protein levels in AFL rats. (E–H) Alcohol‐induced overexpression (E, F) and knockdown (G, H) of CYP1A1 in L02 cells, qPCR, and Western blot analysis of mRNA and protein levels of genes related to the PPARγ pathway. (I–L) Alcohol‐induced overexpression (I, J) and knockdown (K, L) of CYP1A1 in L02 cells, qPCR, and western blot analysis of mRNA and protein levels of genes related to the PPARα pathway. *n* = 3, *p < 0.05, **p < 0.01, ***p < 0.001, ^#^ *p* < 0.05, ^##^ *p* < 0.01.

Alcohol increased PPARγ‐related genes expression (Figure 4A,B, Figure S1A) and decreased PPARα‐related genes expression (Figure 4C,D, Figure S1B), promoting lipid synthesis and inhibiting fatty acid breakdown. Overexpression of CYP1A1 enhanced alcohol‐induced PPARγ‐related genes expression (Figure 4E,F, Figure S1C) and inhibited PPARα‐related genes (Figure 4G,H, Figure S1D), while CYP1A1 knockdown mitigated these effects (Figure 4I–L, Figure 1E,F). In L02 cell, alcohol promotes lipid synthesis and inhibits fatty acid catabolism by up‐regulating PPARγ and down‐regulating PPARα. Overexpression of CYP1A1 further amplifies this PPAR pathway imbalance, thereby exacerbating the accumulation of TC and TG, oxidative stress, and cellular damage. Conversely, CYP1A1 knockdown alleviates these adverse effects.

### 2.4 miR‐18a‐5p Targets CYP1A1 to Inhibit AFL Oxidative Stress

**Figure 5:**
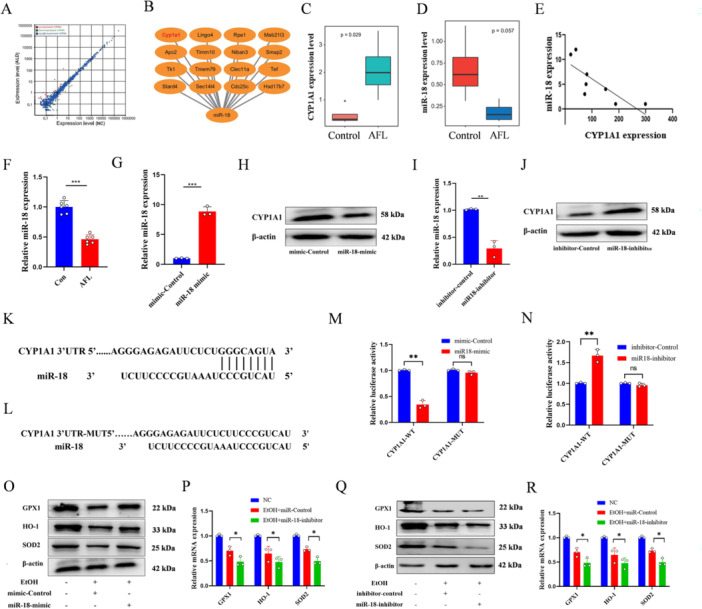
miR‐18a‐5p targets CYP1A1 to ameliorate AFL oxidative stress. (A) Quantitative analysis of miRNA; screening criteria were *p* < 0.05 and |log2FC | > 1. (B) Prediction of miR‐18a‐5p target genes using the Miranda and TargetScan databases. (C, D) Expression levels of CYP1A1 and miR‐18a‐5p in the control and AFL groups. (E) Correlation analysis of CYP1A1 and miR‐18a‐5p expression. (F) qPCR detection of miR‐18a‐5p expression in the control and AFL groups. (G) Transfection efficiency of miR‐18a‐5p mimic. (H) Western blot assessing the impact of miR‐18a‐5p mimic on CYP1A1 protein expression. (I) Transfection efficiency of miR‐18a‐5p inhibitor. (J) Western blot evaluating the effect of miR‐18a‐5p inhibitor on CYP1A1 protein expression. (K) Binding site illustration of miR‐18a‐5p on CYP1A1 3'UTR. (L) Schematic representation of CYP1A1 3'UTR mutation. (M, N) Luciferase reporter assay in L02 cells validating the targeting relationship between miR‐18a‐5p and CYP1A1. (O, P) After transfection with miR‐18a‐5p mimic, western blot and qPCR assessed the transcription of oxidative stress‐related mRNA. (Q, R) Following transfection with miR‐18a‐5p inhibitor, Western blot and qPCR examined the expression of oxidative stress‐related proteins. *n* = 3, *p < 0.05, **p < 0.01, ***p < 0.001.

miRNA sequencing identified 45 differentially expressed miRNAs (Figure 5A, Figure S2A–C), with miR‐18a‐5p negatively correlating with CYP1A1 (Figure 5B–F). miR‐18a‐5p mimic reduced CYP1A1 expression (Figure 5G,H, Figure S2D), while its inhibitor increased it (Figure 5I,J, Figure S2E), confirming miR‐18a‐5p's regulatory role (Figure 5K–N, Figure S2F,G). miR‐18a‐5p ameliorated alcohol‐induced oxidative stress by targeting CYP1A1, enhancing cell viability (Figure S2H,I) and inhibited expression of oxidative stress‐related genes (Figure 5O–R, Figure S2J,K). In summary, miR‐18a‐5p, as a key protective miRNA in AFL, attenuates alcohol‐induced oxidative stress, enhances cell viability, and downregulates related injury genes by targeting and suppressing CYP1A1, thereby providing important evidence for elucidating the early molecular mechanisms of AFL and identifying potential therapeutic targets.

### 2.5 miR‐18a‐5p Targets CYP1A1 via the PPAR Pathway to Ameliorate Lipid Deposition

**Figure 6:**
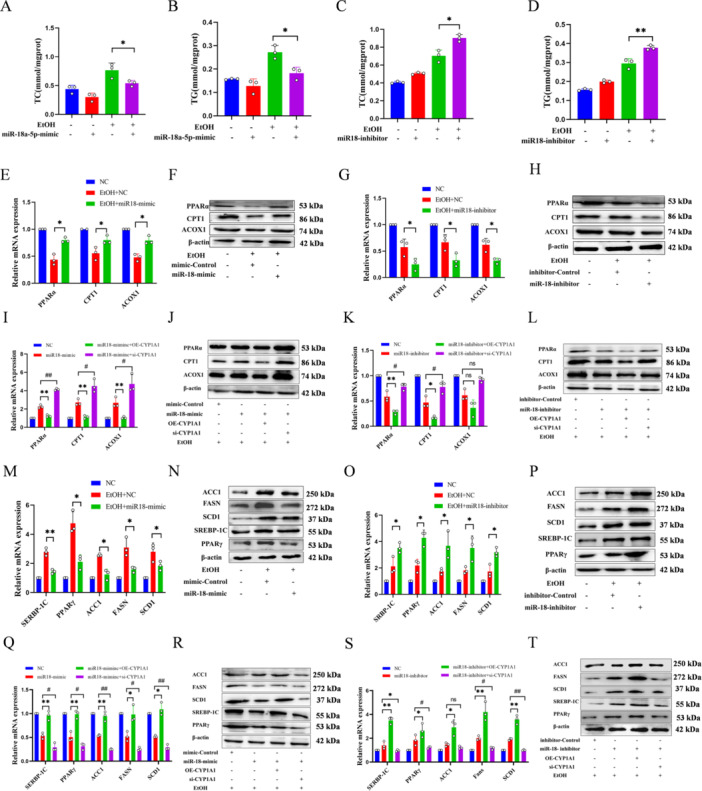
miR‐18a‐5p Targets CYP1A1 to Improve Lipid Deposition via PPAR. (A–D) Transfection with miR‐18a‐5p mimic to assess TC (A) and TG (B) levels in L02 cells, and transfection with miR‐18a‐5p inhibitor to assess TC (C) and TG (D) levels. EtOH: alcohol treatment. (E–L) qPCR and western blot to detect mRNA and protein levels of PPARα‐related genes. miR‐18a‐5p mimic: expression of miR‐18a‐5p; miR‐18a‐5p inhibitor: inhibition of miR‐18a‐5p; OE‐CYP1A1: overexpression of CYP1A1; si‐CYP1A1: knockdown of CYP1A1. (M–T) qPCR and western blot to detect mRNA and protein levels of PPARγ‐related genes. miR‐18a‐5p mimic: expression of miR‐18a‐5p; miR‐18a‐5p inhibitor: inhibition of miR‐18a‐5p; OE‐CYP1A1: overexpression of CYP1A1; si‐CYP1A1: knockdown of CYP1A1. *n* = 3, *p < 0.05, **p < 0.01, ^##^ *p* < 0.01.

**Figure 7:**
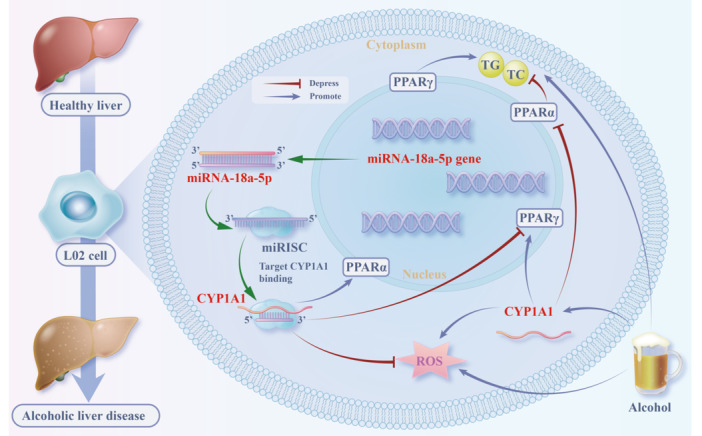
Mechanism of miR‐18a‐5p in suppressing alcoholic fatty liver oxidative stress and lipid deposition via the CYP1A1‐PPAR axis.

miR‐18a‐5p reduced alcohol‐induced TC and TG levels (Figure 6A–D), partially restoring cell viability (Figure S3A,B). It alleviated alcohol‐induced inhibition of PPARα‐related genes (Figure 6E–L, Figure S3C–F) and reduced upregulation of PPARγ‐related genes (Figure 6M–T, Figure S3G–J), improving lipid metabolism through the PPAR pathway by targeting CYP1A1 (Figure 7).

## 3 Discussion

Our study elucidates the regulatory role of the miR‐18a‐5p/CYP1A1/PPAR axis in AFL. Mechanistically, miR‐18a‐5p directly targets CYP1A1 to suppress its expression, thereby mitigating oxidative stress and modulating PPARα/γ‐related gene expression—ultimately reducing lipid deposition and improving AFL outcomes. This regulatory cascade provides novel insights into AFL pathogenesis and potential therapeutic targeting.

The progression of AFL disease is closely related to mechanisms such as lipid accumulation, insulin resistance, oxidative stress, and inflammation. Both alcoholic and non‐AFL diseases share similar pathological pathways [17, 18, 19, 20, 21, 22, 23]. Progression to hepatocellular carcinoma carries high mortality, with 15% of patients succumbing to organ shortage during liver transplantation waiting periods [24], underscoring the urgency to identify actionable targets. Through transcriptome sequencing of AFL rat models, we identified differentially expressed genes enriched in oxidative stress, lipid metabolism, and PPAR signaling pathways. Cross‐referencing with online databases pinpointed CYP1A1—a CYP450 family member involved in xenobiotic and endogenous metabolism [25]—as a key candidate. Concurrent miRNA sequencing revealed 45 differentially expressed miRNAs linked to oxidative stress and lipid metabolism, with miR‐18a‐5p emerging as a negative regulator of CYP1A1 via direct 3'UTR binding. These findings lend credence to the common mechanisms at the basis of alcoholic and non‐AFL, suggesting that these two entities have much more similar pathways than thought before [26].

The PPAR family, a group of ligand‐dependent transcription factors, plays a central role in hepatic metabolic regulation [27]. PPARγ, abundantly expressed in adipose tissue, contributes to lipid metabolism [28, 29], and its ethanol‐induced upregulation—mediated by ABL2‐HIF1α signaling—promotes lipid deposition in ALD [30]. Conversely, PPARα, critical for fatty acid homeostasis and inflammation control, exhibits reduced activity/expression in ethanol‐exposed mouse livers, a key driver of steatosis, hepatitis, and fibrosis. Ethanol‐induced suppression of PPARα transcriptional activity is reversed by PPARα agonists, which protect against lipid accumulation and inflammation [31, 32, 33]. Our findings extend these observations by demonstrating that ethanol synergistically upregulates fatty acid synthesis genes while downregulating PPARα‐dependent fatty acid/cholesterol catabolic genes, creating a metabolic imbalance that exacerbates lipid deposition.

CYP1A1 has been implicated in non‐alcoholic fatty liver disease (NAFLD) progression via oxidative stress [34], lipid deposition [35], and inflammation [36]. However, its role in AFL remains understudied. Our data support a pro‐AFL function for CYP1A1: in both AFL rats and in vitro models, CYP1A1 exacerbates oxidative stress and lipid deposition through PPAR pathway modulation. This contrasts with NAFLD mechanisms but aligns with reports that CYP1A1 is a PPAR target—serum‐induced CYP1A1 expression via PPARα [37] and upregulated CYP1A1 activity by PPARα/γ agonist [38]—suggesting a positive feedback loop between CYP1A1 and PPAR that accelerates AFL pathogenesis.

Although existing studies have demonstrated the role of miRNA in NAFLD [39]. In the present study, we found that miR‐18a‐5p emerges as a critical upstream regulator. By targeting CYP1A1, miR‐18a‐5p enhances PPARα‐related gene expression, inhibits PPARγ upregulation, reduces oxidative stress, and improves cell viability—collectively ameliorating alcohol‐induced lipid deposition. This aligns with broader evidence that miRNAs modulate hepatic lipid metabolism: for example, miR‐18a‐5p has been linked to lipid homeostasis in other metabolic disorders, while related miRNAs (e.g., miR‐122) regulate PPAR signaling in liver disease [40]. However, our study is the first to identify miR‐18a‐5p as a specific regulator of the CYP1A1/PPAR axis in AFL, distinguishing it from miR‐122, which primarily targets SREBP‐1c in ethanol‐induced steatosis.

Limitations of this study should be acknowledged. First, our conclusions are based solely on animal (rat) and cell culture models; validation in human AFL samples is required to confirm the clinical relevance of the miR‐18a‐5p/CYP1A1/PPAR axis. Second, the molecular mechanisms underlying the CYP1A1‐PPAR feedback loop—including potential cofactors or posttranslational modifications—remain underexplored. Third, we did not investigate off‐target effects of miR‐18a‐5p, which may influence other pathways in AFL pathogenesis.

Future research directions should focus on three areas: Clinical validation: assessing miR‐18a‐5p and CYP1A1 expression patterns in human AFL biopsies to correlate with disease severity. Mechanistic refinement: exploring epigenetic regulation of the CYP1A1‐PPAR loop and identifying additional miR‐18a‐5p targets in AFL. Translational potential: developing miR‐18a‐5p mimics or CYP1A1 inhibitors for preclinical testing, with a focus on delivery systems targeting hepatocytes. Successful translation could provide a novel therapeutic strategy to halt AFL progression and reduce the burden of end‐stage liver disease.

In summary, our study identifies miR‐18a‐5p as a key regulator of the CYP1A1/PPAR axis in AFL, with therapeutic potential to mitigate oxidative stress and lipid deposition. These findings bridge knowledge gaps in miRNA‐mediated metabolic regulation in ALD and lay the groundwork for future translational research.

## 4 Methods

### 4.1 Ethics Statement

All animal procedures were carried out in accordance with the regulations for the administration of affairs concerning experimental animals, which compliant the ARRIVE guidelines and US National Institutes of Health Guide for the Care and Use of Laboratory Animals. All experiments were approved by the Jiamusi University Affiliated First Hospital Medical Ethics Committee (2022‐500‐34). According to these guidelines, efforts were made to minimize the number of animals used and their suffering.

### 4.2 Establishment of the AFL Rat Model and Serum Analysis

Twenty male specific pathogen‐free (SPF) Sprague‐Dawley (SD) rats, between 180 and 220 grams weight, were used in this study. The rats were acclimated for 1 week before experiments. They were assigned at random to two groups: the control group (*n* = 10) and the alcohol‐fed group (*n* = 10), and other experiments are performed with three independent repetitions. The control group received twice‐daily gavage of physiological saline, while the alcohol‐fed group received alcohol gavage with a stepwise increasing concentration for 12 weeks. From Week 1 to 4, 8 mL·kg⁻¹·d⁻¹ of 30% alcohol was administered daily by gavage; from Week 5 to 8, the dosage was increased to 8 mL·kg⁻¹·d⁻¹ of 35% alcohol; and from Week 9 to 12, 8 mL·kg⁻¹·d⁻¹ of 40% alcohol was administered daily by gavage. Body weight was measured weekly.

After an overnight fasting, rats were intraperitoneally injected with 30 mg/kg pentobarbital sodium for euthanasia. Blood was collected from the abdominal aorta and analyzed for alanine transaminase (ALT), aspartate aminotransferase (AST), TNF‐α, interleukin (IL)−1β, free fatty acids (FFA), total cholesterol (TC), and triglycerides (TG) using commercially available kits following the manufacturer's protocols.

### 4.3 Histological Examination

Liver tissues were harvested and preserved in 10% neutral‐buffered formalin. They were then embedded in paraffin and sectioned into 5 µm slices. Sections were stained with hematoxylin and eosin (H&E) for histological examination. Liver tissue sections were also analyzed for lipid content using Oil Red O staining.

### 4.4 RNA Extraction and Sequencing

Total RNA was extracted from liver tissues using TRIzol reagent according to the manufacturer's instructions. RNA integrity was evaluated using the Agilent 2100 Bioanalyzer. RNA sequencing (RNA‐seq) was conducted on an Illumina HiSeq. 2500 platform. Differentially expressed genes (DEGs) were identified using DESeq. 2 software, with a threshold of an adjusted *p* < 0.05 and a fold change > 2.

### 4.5 Bioinformatic Analysis

To identify enriched biological processes and pathways among the DEGs, Gene Ontology (GO) and Kyoto Encyclopedia of Genes and Genomes (KEGG) pathway analyses were conducted. The Gene Expression Omnibus (GEO) database was utilized to cross‐reference our findings with existing datasets. Differential gene protein‐protein interaction (PPI) network analysis was conducted using the STRING database, and the core protein sub‐network analysis was performed using the GeneMANIA database. Bioinformatics analysis was visualized using the Microbio website (http://www.bioinformatics.com.cn/). Prediction of miRNA target genes was carried out using the Miranda and TargetScan databases.

### 4.6 Quantitative PCR (qPCR) Validation

cDNA was produced from total RNA using a reverse transcription kit. qPCR was undertaken with SYBR Green Master Mix on an ABI 7500 Fast Real‐Time PCR System. The relative expression levels of genes were normalized to GAPDH and calculated using the 2^−ΔΔCt method. Primer sequences (Sangon Biotech, Shanghai Co. Ltd.) were listed in Table S1.

### 4.7 Western Blotting (WB)

Cell lysis was conducted using RIPA buffer (supplemented with phenylmethylsulphonyl fluoride [PMSF]) to extract total proteins. Protein concentrations were determined using a BCA assay. For Western blot analysis, 30 μg of total protein per sample was loaded onto 10% polyacrylamide gels for electrophoresis. Separated proteins were transferred to polyvinylidene difluoride (PVDF) membranes. Following blocking with 5% nonfat milk for 1 h at room temperature, the membranes were incubated overnight at 4°C with respective primary antibodies (1:1000 dilution; rabbit anti‐human antibodies against layer protein A, layer protein C, β‐actin [Abcam, USA], p16, CDK1, MMP2, and MMP9 [BOSTER, China]). Membranes were then incubated with secondary antibodies (1:8000 dilution, mouse anti‐rabbit IgG [Abcam, USA]) for 1 h at room temperature. Protein bands were visualized using a gel imaging system, and band intensities were quantified via densitometry using ImageJ software (Version 1.53e), with β‐actin serving as the internal reference.

### 4.8 Cell Culture and Treatment

The human hepatic cell line L02 cells and HEK293T cell line were purchased from Procell Technology Co. Ltd. (Wuhan, China). The cells were cultured in Dulbecco's Modified Eagle Medium (DMEM) supplemented with 10% fetal bovine serum (FBS) and incubated at 37°C in a 5% CO_2_ atmosphere. Cells were treated with varying concentrations of ethanol to establish an in vitro model of AFL.

### 4.9 Transfection and Luciferase Reporter Assays

Cells were transfected with miR‐18a‐5p mimic, inhibitor, or negative control using Lipofectamine 2000 following the manufacturer's protocol. Wild‐type and mutant CYP1A1 3’‐UTR sequences were then cloned into the pGL3 luciferase reporter vector for luciferase reporter assays. Luciferase activity was assessed 48 h after transfection using the Dual‐Luciferase Reporter Assay System.

### 4.10 ROS Detection

After removing the treated cell culture medium, cells were dissociated into single cells using trypsin. Dichlorodihydrofluorescein diacetate (DCFH‐DA) was diluted in serum‐free culture medium at a 1:1000 ratio to achieve a 10 μM final concentration, and diluted DCFH‐DA was added at an appropriate volume. Cells were then incubated at 37°C for 20 min to allow DCFH‐DA uptake, followed by three washes with serum‐free medium to remove excess DCFH‐DA. Intracellular ROS levels were measured using flow cytometry.

### 4.11 Cell Counting Kit‐8 (CCK‐8)

Cells in the logarithmic growth phase were seeded at a density of 3000 cells per well in a 96‐well plate and incubated overnight in a cell culture incubator. The next day, cells were either transfected or treated with varying concentrations of alcohol. Following transfection or alcohol treatment, cells were further incubated for 24 to 48 h. Subsequently, 10 µL of CCK‐8 solution was added to each well, and after incubating at 37°C for 1 h, absorbance at 450 nm was measured using a microplate reader.

### 4.12 Measurement of Oxidative Stress Markers

Malondialdehyde (MDA) levels, glutathione (GSH) levels, and superoxide dismutase (SOD) activity in serum samples and cell lysates were measured using commercially available assay kits.

### 4.13 Statistical Analysis

All data are expressed as mean ± standard deviation (SD). Statistical analyses were performed using GraphPad Prism 8.0 software. Differences between groups were evaluated using Student's *t*‐test or one‐way ANOVA followed by Tukey's post‐hoc test. A *p*‐value < 0.05 was considered statistically significant.

## 5 Conclusions

Our study proposes the miR‐18a‐5p/CYP1A1/PPAR axis as a novel pathway in AFL. miR‐18a‐5p targets CYP1A1, reducing its expression and mitigating oxidative stress and lipid deposition through the PPAR pathway. This axis could serve as a promising therapeutic target for AFL treatment.

## Author Contributions

**XueMei Zhang:** conceptualization, validation. **Wei Li:** software, validation. **Ubaid Ullah:** methodology. **Ning Li:** formal analysis. **XinYu Geng:** resources. **JiHan Qi:** visualization. **HongLiang Chen:** supervision. **Xu Zhang:** resources. **Ying Hu:** data curation. **Lingling Yang:** resources. **ShiZhu Jin:** conceptualization, writing review and editing, project administration, funding acquisition. All authors have read and agreed to the published version of the manuscript.

## Conflicts of Interest

The authors declare no conflicts of interest.

## Supporting information

Supportingfigure1.

Supportingfigure2.

Supportingfigure3.

Supporting for Figure4.

Supporting for Figure5.

Supporting for Figure6.

Supporting Table 1.

## Data Availability

The data sets analyzed during this study are available from the GEO with accession number GSE28619. This data can be found here: https://www.ncbi.nlm.nih.gov/geo/query/acc.cgi?acc=GSE28619. All the data that support the conclusions of this study can be retrieved from the corresponding author (drshizhujin@hrbmu.edu.cn).
