# Supplementary figures and images for: MiR‐18a‐5p Attenuates Oxidative Stress and Inhibits Lipid Accumulation in Alcoholic Fatty Liver by Activating the CYP1A1‐PPAR Axis
Source: Immun Inflamm Dis. 2026 Feb 5;14(2):e70350. doi: 10.1002/iid3.70350 (PMC12877320; doi:10.1002/iid3.70350)

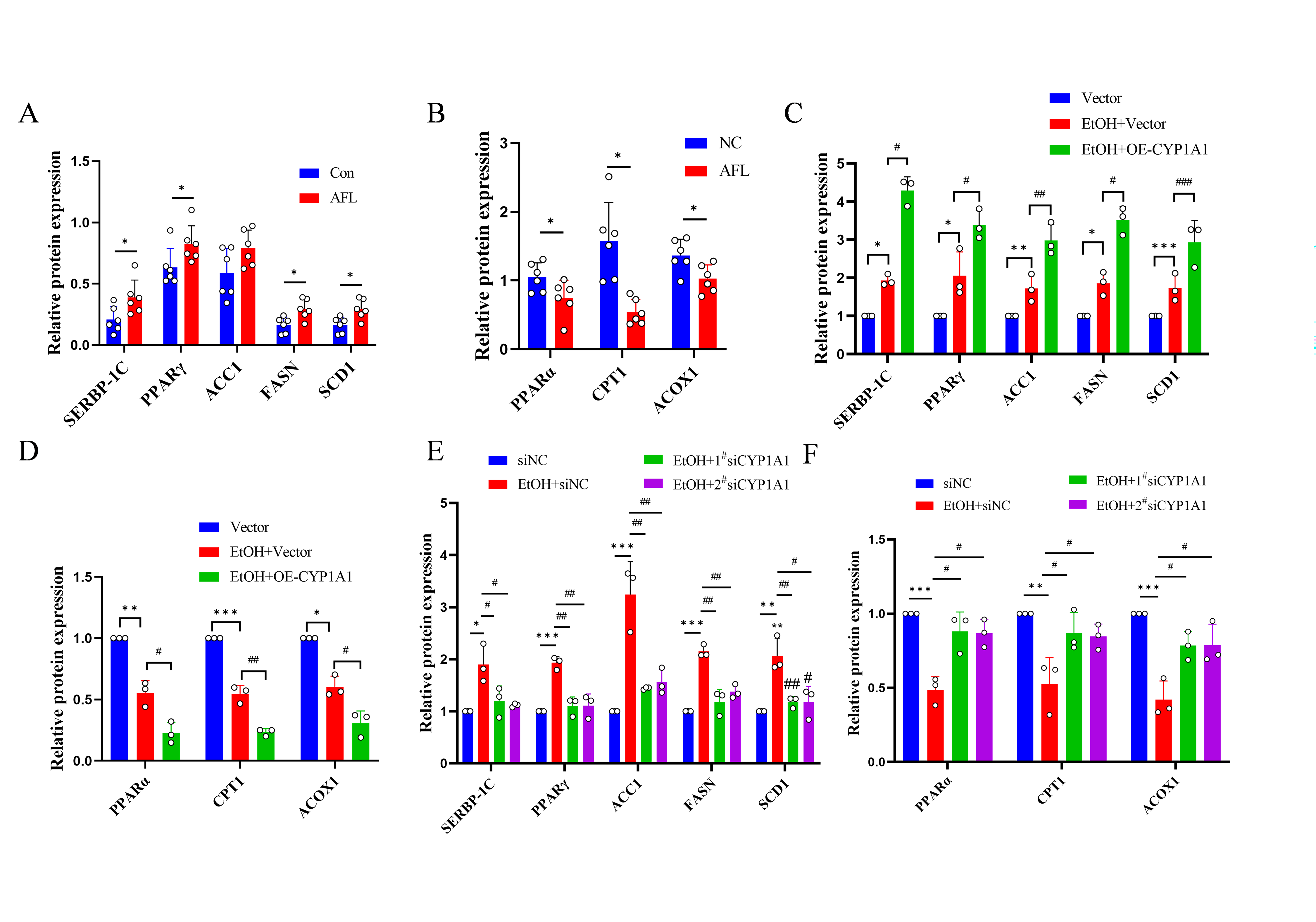

Supplement: Supplementary file 1 — Supportingfigure1. [file IID3-14-e70350-s005.tif]

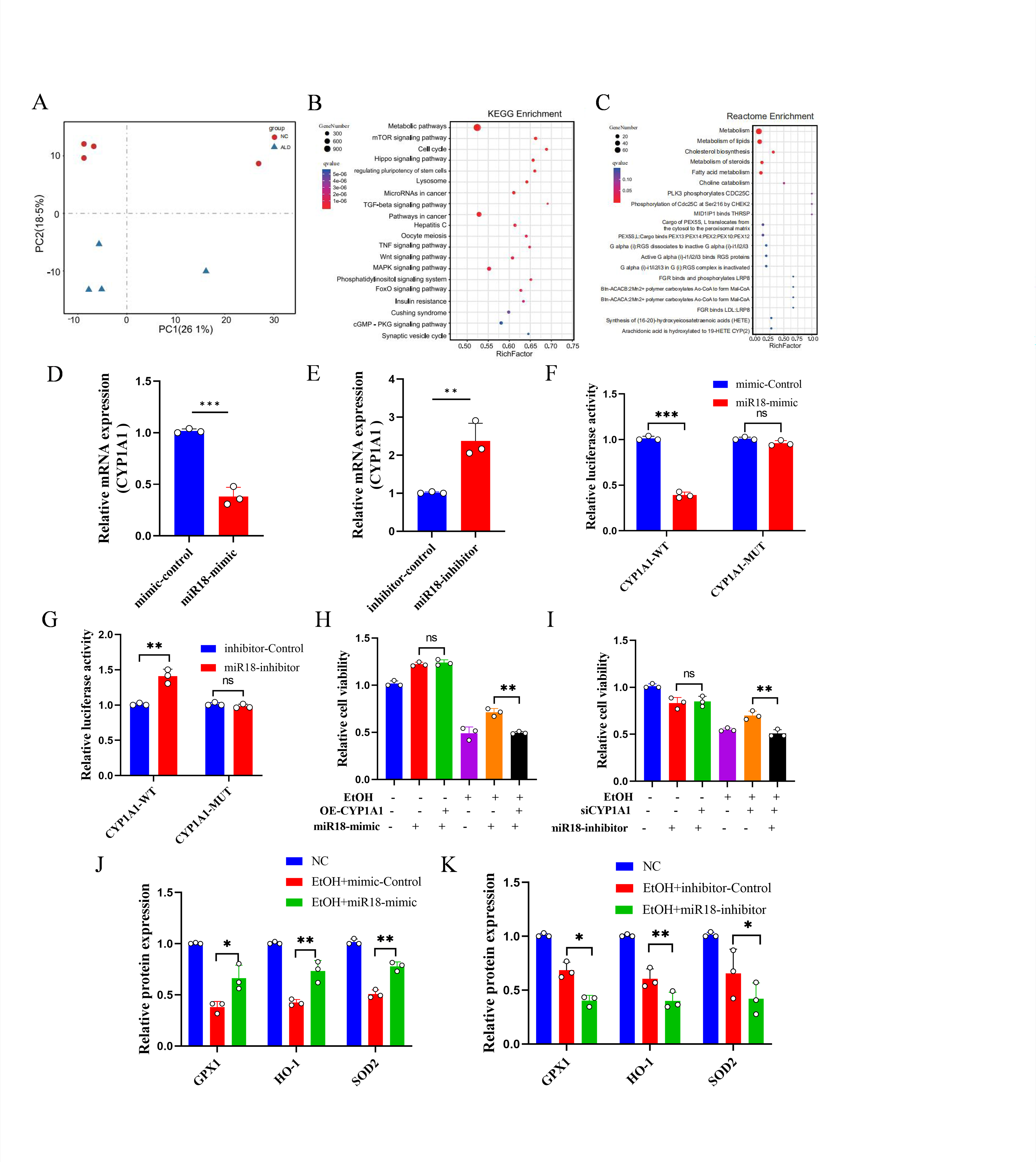

Supplement: Supplementary file 2 — Supportingfigure2. [file IID3-14-e70350-s006.tif]

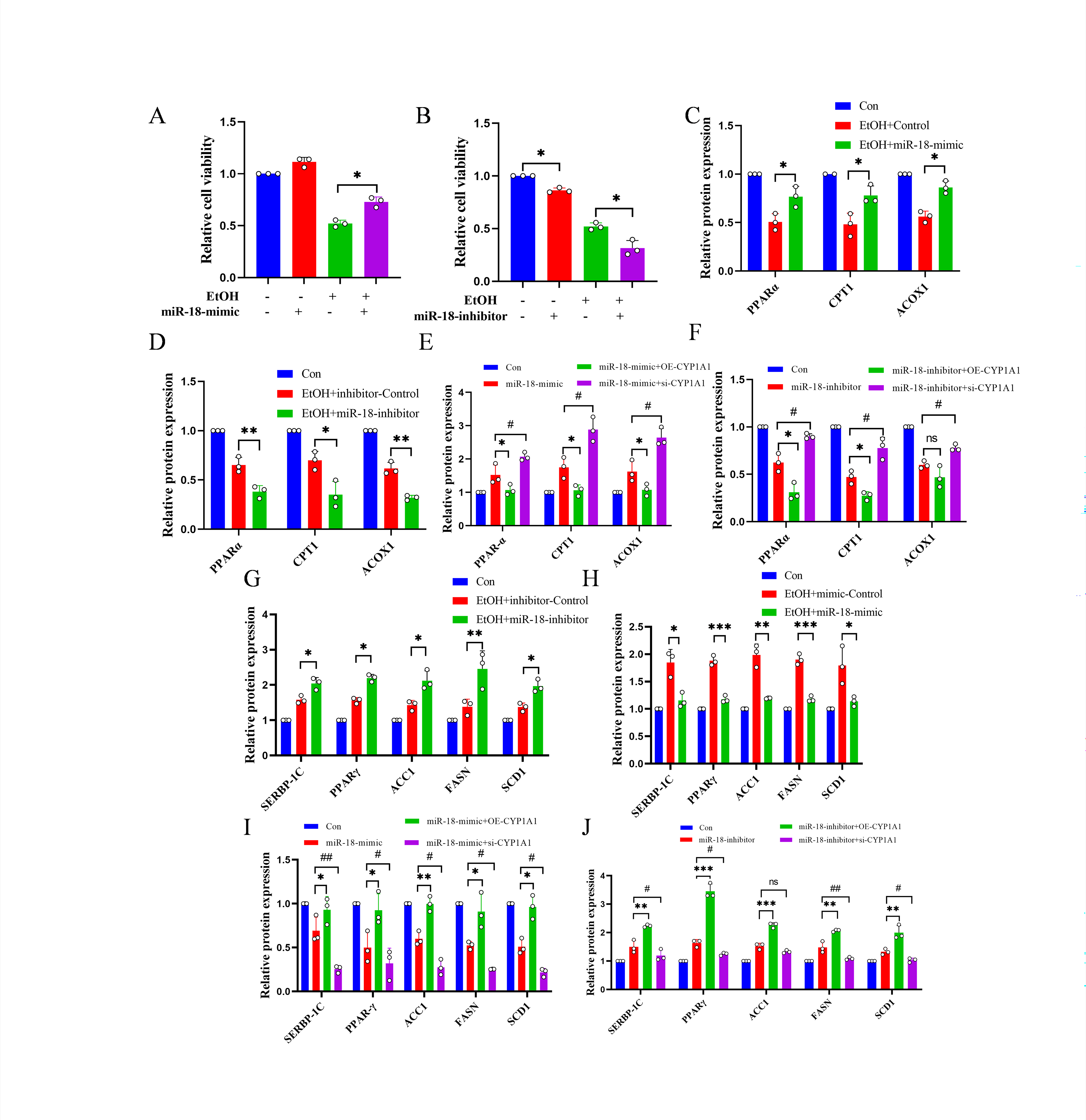

Supplement: Supplementary file 3 — Supportingfigure3. [file IID3-14-e70350-s004.tif]

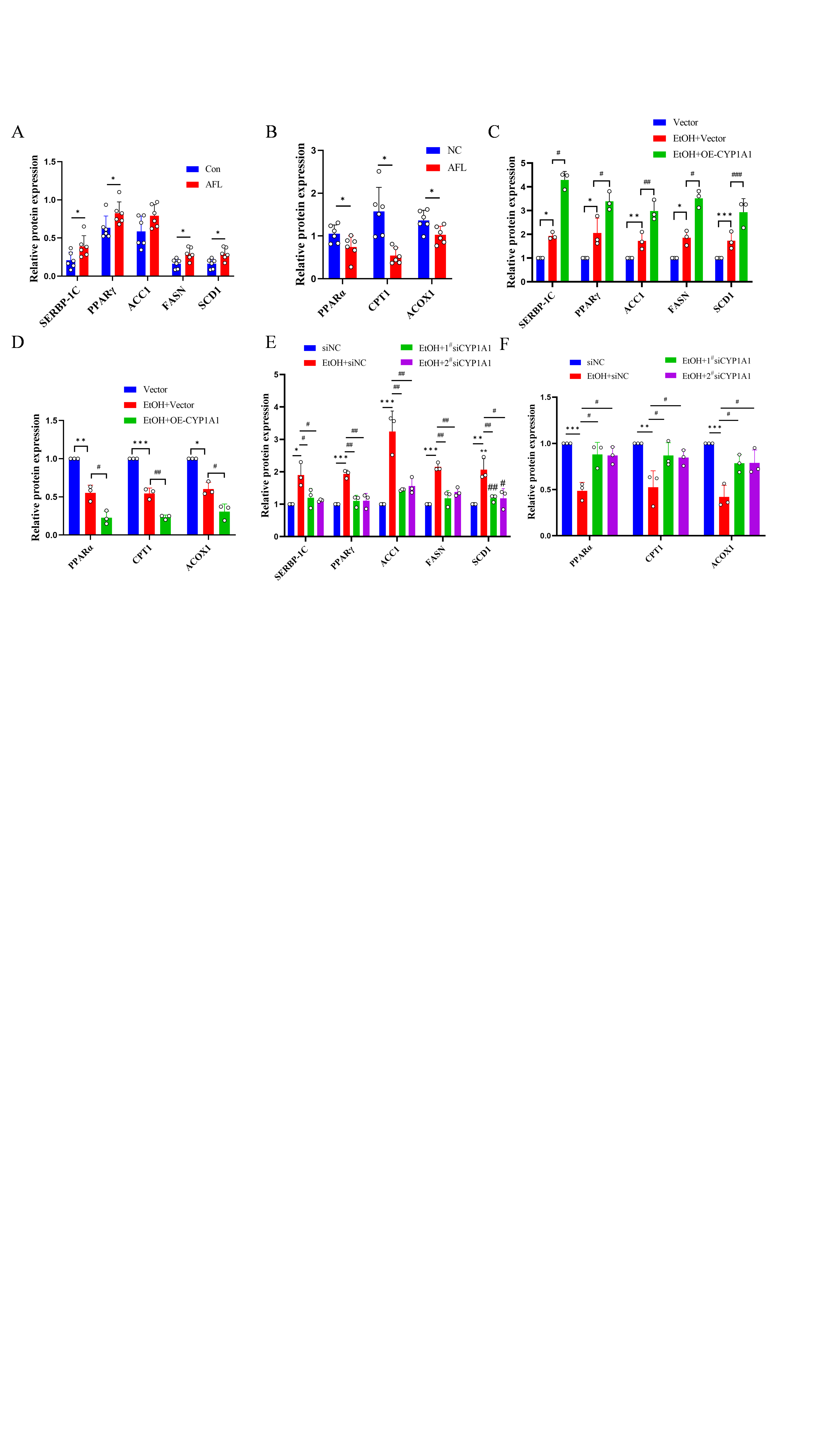

Supplement: Supplementary file 4 — Supporting for Figure4. [file IID3-14-e70350-s007.tif]

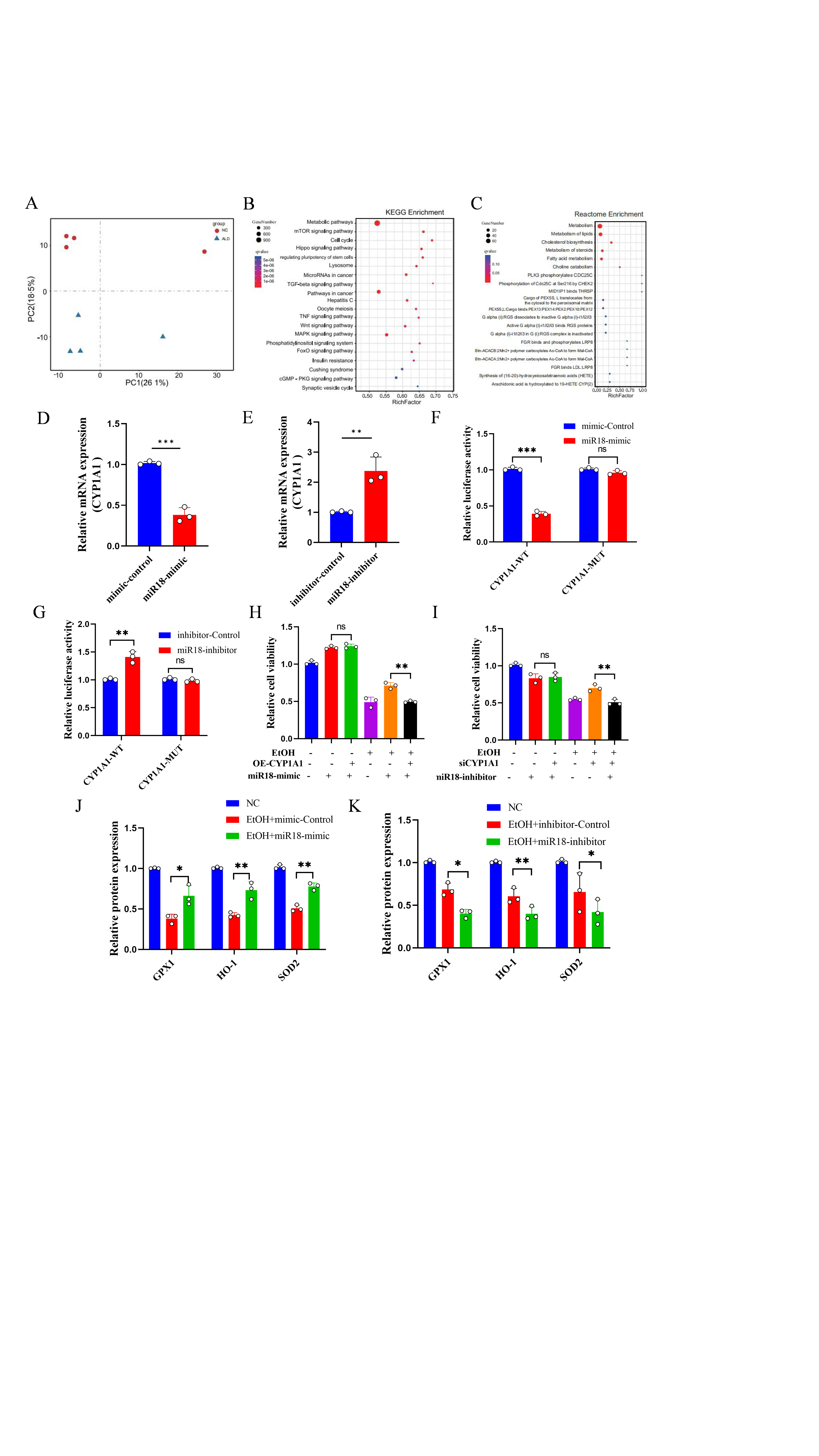

Supplement: Supplementary file 5 — Supporting for Figure5. [file IID3-14-e70350-s002.tif]

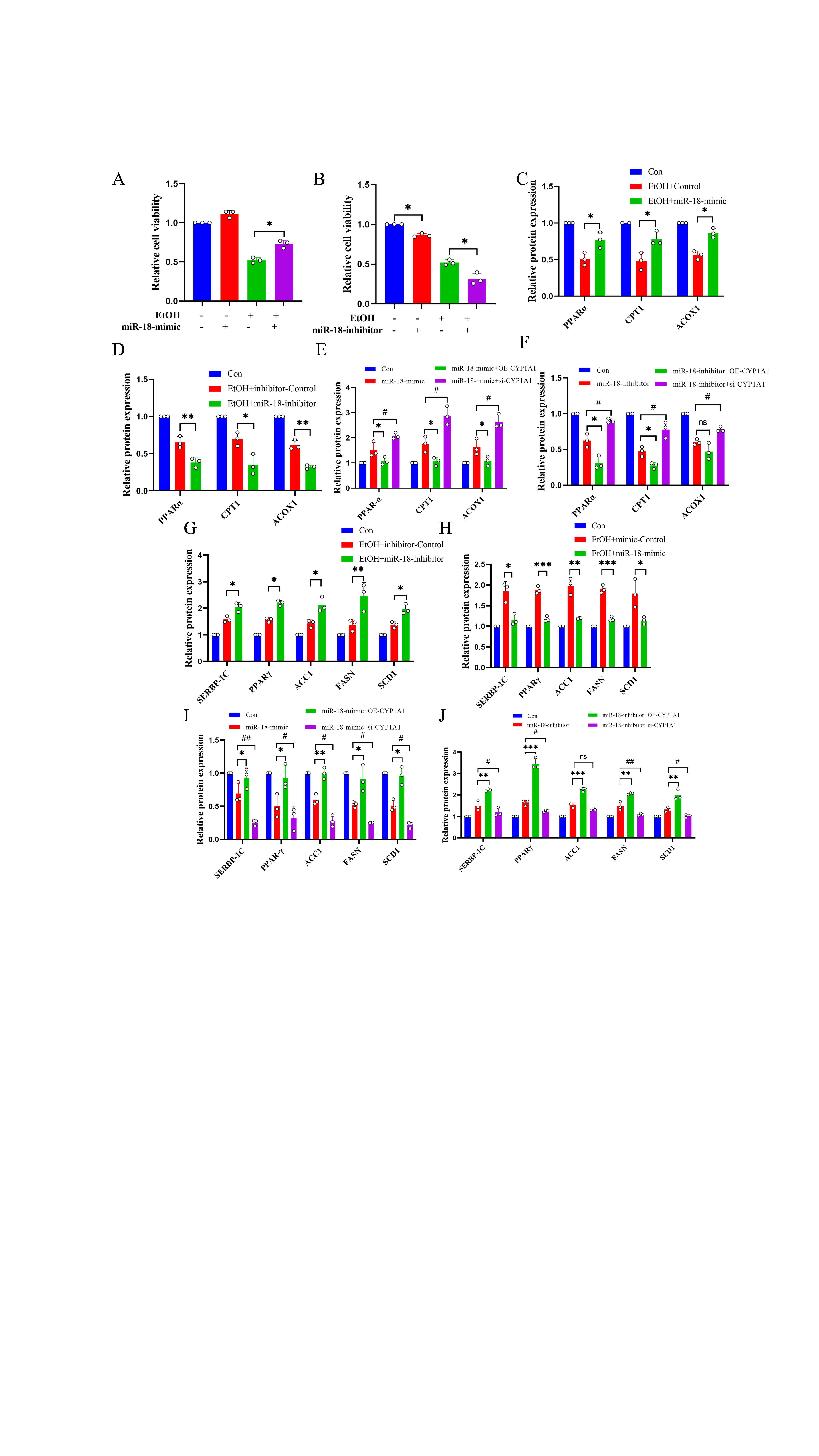

Supplement: Supplementary file 6 — Supporting for Figure6. [file IID3-14-e70350-s003.tif]
